# Predictors for timely initiation of breastfeeding after birth in the hospitals of Nepal- a prospective observational study

**DOI:** 10.1186/s13006-021-00431-y

**Published:** 2021-10-29

**Authors:** Rejina Gurung, Avinash K. Sunny, Prajwal Paudel, Pratiksha Bhattarai, Omkar Basnet, Srijana Sharma, Durgalaxmi Shrestha, Seema Sharma, Honey Malla, Dela Singh, Sangeeta Mishra, Ashish KC

**Affiliations:** 1 Golden Community, Lalitpur, Nepal; 2 Department of Women’s and Children’s Health, Uppsala University, Dag Hammarskjöldsväg 14B, Uppsala, Sweden; 3 Paropakar Maternity and Women’s Hospital, Kathmandu, Nepal; 4 Bheri Provincial Hospital, Nepalgunj, Nepal; 5 Seti Provincial Hospital, Dhangadi, Nepal; 6 Pokhara Academy of Health Sciences, Pokhara, Nepal

**Keywords:** Predictor, Timely initiation of breastfeeding, Neonate placed skin to skin contact, Delayed cord clamping, Nepal

## Abstract

**Background:**

Timely initiation of breastfeeding can reduce neonatal morbidities and mortality. We aimed to study predictors for timely initiation of breastfeeding (within 1 h of birth) among neonates born in hospitals of Nepal.

**Method:**

A prospective observational study was conducted in four public hospitals between July and October 2018. All women admitted in the hospital for childbirth and who consented were included in the study. An independent researchers observed whether the neonates were placed in skin-to-skin contact, delay cord clamping and timely initiation of breastfeeding. Sociodemographic variables, obstetric and neonate information were extracted from the maternity register. We analysed predictors for timely initiation of breastfeeding with Pearson chi-square test and multivariate logistic regression.

**Results:**

Among the 6488 woman-infant pair observed, breastfeeding was timely initiated in 49.5% neonates. The timely initiation of breastfeeding was found to be higher among neonates who were placed skin-to-skin contact (34.9% vs 19.9%, *p* - value < 0.001). The timely initiation of breastfeeding was higher if the cord clamping was delayed than early cord clamped neonates (44.5% vs 35.3%, *p* - value < 0.001). In multivariate analysis, a mother with no obstetric complication during admission had 57% higher odds of timely initiation of breastfeeding (aOR 1.57; 95% CI 1.33, 1.86). Multiparity was associated with less timely initiation of breastfeeding (aOR 1.56; 95% CI 1.35, 1.82). Similarly, there was more common practice of timely initiation of breastfeeding among low birthweight neonates (aOR 1.46; 95% CI 1.21, 1.76). Neonates who were placed skin-to-skin contact with mother had more than two-fold higher odds of timely breastfeeding (aOR 2.52; 95% CI 2.19, 2.89). Likewise, neonates who had their cord intact for 3 min had 37% higher odds of timely breastfeeding (aOR 1.37; 95% CI 1.21, 1.55).

**Conclusions:**

The rate of timely initiation of breastfeeding practice is low in the health facilities of Nepal. Multiparity, no obstetric complication at admission, neonates placed in skin-to-skin contact and delay cord clamping were strong predictors for timely initiation of breastfeeding. Quality improvement intervention can improve skin-to-skin contact, delayed cord clamping and timely initiation of breastfeeding.

## Background

Envisioning a world where every child can survive and thrive requires global commitment to promote and support evidence-based neonatal and child health interventions [1]. Worldwide, the neonatal mortality rate has fallen with a decrease in neonatal sepsis which is a major killer disease [2]. A systematic review has shown that the early initiation of breastfeeding has reduced neonatal mortality due to any cause among all live births by 44% and mortality among low birthweights by 42% and infection related mortality by 45% [3]. The World Health Organization recommends breastfeeding to be initiated within 1 h of birth and to be provided exclusively till 6 months of age [4, 5]. Provision of immediate newborn care that encompasses timely initiation of breastfeeding within 1 h of birth is crucial to accelerate survival [6].

In 2016, only about 55% of the mothers initiated timely breastfeeding in Nepal [7]. Antenatal visit, obstetric complication and mode of delivery was associated with timely initiation of breastfeeding [8–10]. Women who delivered at home had a higher rate of timely initiation of breastfeeding than those who delivered at health facility [11]. A multi-country health facility-based observation study including Nepal as a site showed that only 10.9% women timely initiated breastfeeding in health facilities [12]. The neonatal factors such as sex of the neonate, Apgar score and gestational age were associated with timely initiation of breastfeeding [13]. Immediate newborn care practice such as placing baby on mother’s chest immediately after the birth had association with timely initiation of breastfeeding [14–16].

In Nepal, efforts have been made to promote breastfeeding in health facilities through implementation of BFHI (Baby Friendly Hospital Initiative) and Community Based Newborn Care Package (CB-NCP) [17, 18]. Considering the coverage of institutional deliveries in Nepal by 60%, timely initiation of breastfeeding is relatively low; raising a concern to scale up the intervention [7]. In this study, we aim to identify predictors for timely initiation of breastfeeding in health facilities in Nepal.

## Methods

### Study design and setting

A prospective observational study was conducted in four public hospitals of Nepal between 1 July 2017 and 17 October 2018 [19]. Koshi Hospital, Bharatpur Hospital, Lumbini Hospital and Western Regional hospital were selected for this particular study.

### Study participants

All pregnant women admitted for vaginal delivery were eligible for the study. Those mothers undergoing caesarean birth, stillbirths and those who did not consent or avail themselves for interview were excluded from the study.

### Data collection and management

Data were collected using paper-based forms through a data surveillance system established in all four hospitals. An eligible pregnant woman admitted to hospital for delivery was enrolled in the study after obtaining her written informed consent. The pregnant woman was then tracked for the observation of intrapartum care received by the mother including immediate newborn care. Twenty-four-hour observation was done by a team of surveillance officers. Sociodemographic, obstetric and newborn’s general information were extracted from patient’s chart. Both extraction and observation forms were then assessed for completeness by data coordinator placed in each hospital. A weekly surveillance form was filled by the data coordinator. At the end of every week all these filled forms were sent to the central data management office in a sealed envelope. Data were then entered into database by a team of data entry operator using the Census and Survey Processing System (CSPro).

### Study variables

#### Obstetric variables

Parity categorized as nullipara (no previous birth), primipara (one previous birth) and multipara (2–5 previous births) [12]. Obstetric complications at admission were antepartum hemorrhage, hypertensive disorder during pregnancy, premature rupture of membrane, cord prolapse and infection. Mode of birth categorized as spontaneous vaginal and instrumental delivery [12]. Gestational age categorized as < 37 weeks, 37–41 weeks and ≥ 42 weeks. Birthweight categorized as ≥2500 and < 2500 g. Early initiation of breastfeeding defined as neonate breastfed within 1 h of birth [12]. Delayed cord clamping defined as cord clamped at 3 min or more. Immediate skin to skin contact defined as neonates placed skin to skin contact with mother soon after birth [12].

#### Outcome variables

Timely initiation of breastfeeding was defined as the proportion of neonates who were breastfed within the first hour of birth.

### Statistical analysis

Data were exported into Statistical Package for Social Sciences (SPSS) version 23 for analysis. Pearson chi-square test was used to find out the association between various variables with timely breastfeeding initiation and multiple logistic regression was used to analyze the adjusted odds of the association. At 95% Confidence Interval, *p* - value < 0.05 was considered to be statistically significant. Missing data were excluded from the analyses.

## Results

**Fig. 1:**
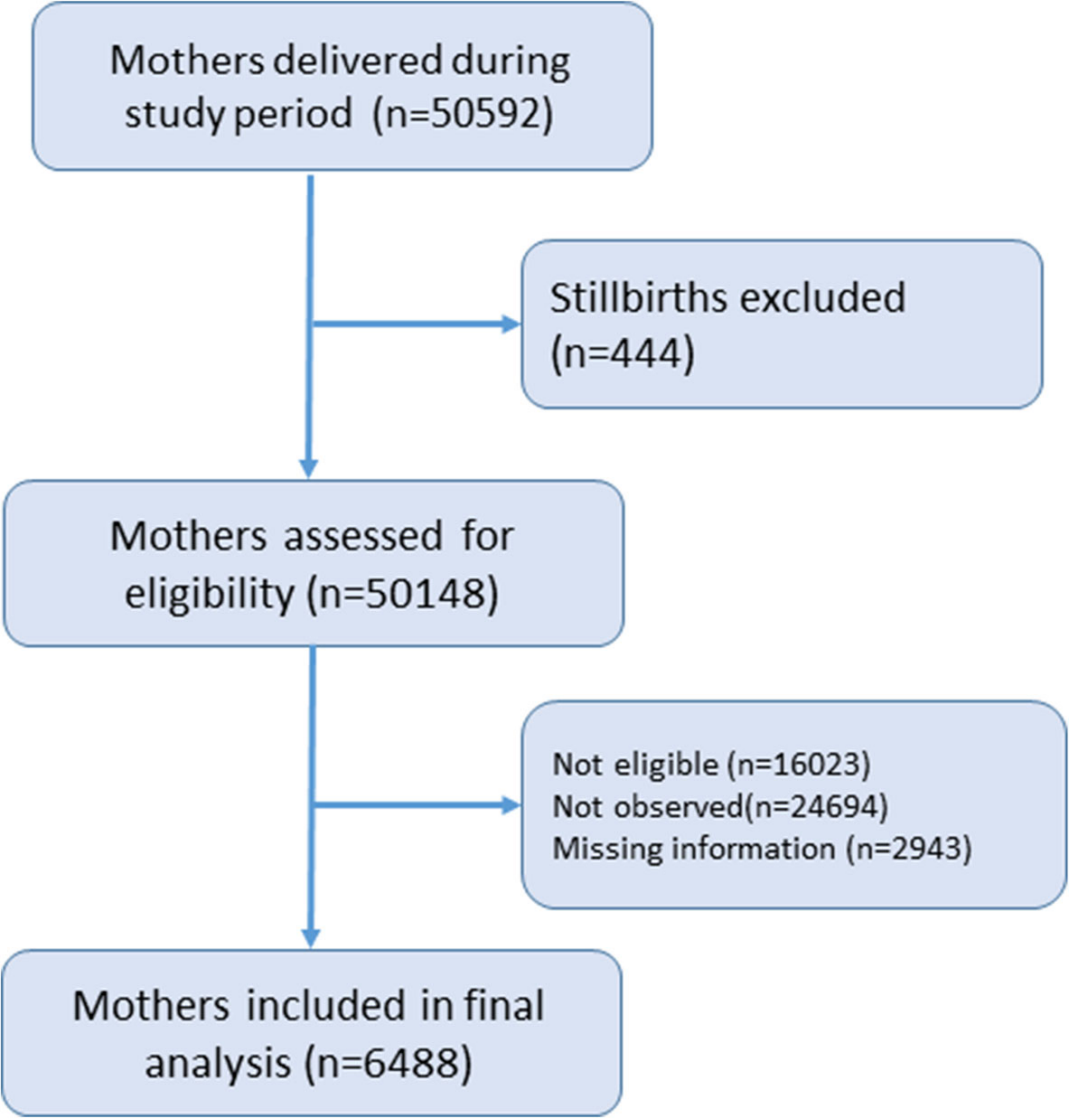
Study flow diagram

During the study period, total of 6488 mothers were observed for breastfeeding, among whom 49.5% of them had timely breastfeeding (Fig. 1). During the study period, 27.3% of neonates were placed skin-to-skin contact with mother soon after birth and 39.5% of neonates had delay cord clamping (Table 1).

**Table 1:** Hospital wise coverage of timely initiation of breastfeeding, neonates placed immediately in skin to skin contact with mother and delayed cord clamping (*n* = 6488)

Hospitals	Timely initiation of breastfeeding		Neonates placed immediately in skin to skin contact with mother		Delayed cord clamping	
	***N***	% (95% CI)	***N***	% (95% CI)	***N***	% (95% CI)
Western Regional Hospital	2358/3917	60.2 (58.7, 61.7)	329/3913	8.4 (7.5, 9.3)	1156/2671	43.3 (41.4, 45.2)
Bharatpur Hospital	64/752	8.5 (6.5, 10.5)	124/752	16.5 (13.8, 19.1)	196/741	26.5 (23.3, 29.6)
Koshi Hospital	6/450	1.3 (0.3, 2.4)	38/450	8.4 (5.9, 11.0)	54/448	12.1 (9.0, 15.1)
Lumbini Hospital	782/1369	57.1 (54.5, 59.7)	1282/1369	93.6 (92.4, 94.9)	656/1354	48.4 (45.8, 51.1)
Total	3210/6488	49.5 (48.3, 50.7)	1773/6484	27.3 (26.3, 28.4)	2062/5214	39.5 (38.2, 40.9)

The timely initiation of breastfeeding was higher among women who had one previous birth compared to women who didn’t have (37.8% vs 28.8%, *p* - value < 0.001). The practice was more common among women with no obstetric complication at the time of admission (88.7% vs 11.3%, *p* - value < 0.001). Timely initiation of breastfeeding was high among babies who were placed in skin to skin contact (34.9%, *p* - value < 0.001). There was higher coverage of timely initiation of breastfeeding in delay cord clamped neonates than in early clamped (35.3% vs 44.5%, *p* - value < 0.001) (Table 2).

**Table 2:** Coverage of timely initiation of breastfeeding (*n* = 6488)

Variables	Timely initiation of breastfeeding			***p***-value	cOR (95%CI)
	No	Yes	Total		
**Parity**				< 0.001	
1 previous birth	1359 (41.5%)	1214 (37.8%)	2573 (39.7%)		Ref
0 previous birth	1084 (33.1%)	923 (28.8%)	2007 (30.9%)		0.95 (0.85, 1.07)
2 or more previous births	835 (25.5%)	1072 (33.4%)	1907 (29.4%)		1.44 (1.28, 1.62)
**Obstetric Complication**				< 0.001	
Yes	643 (20.6%)	346 (11.3%)	989 (16.0%)		Ref
No	2483 (79.4%)	2721 (88.7%)	5204 (84.0%)		2.04 (1.77, 2.35)
**Mode of delivery**				0.34	
Spontaneous vaginal delivery	3163 (96.5%)	3083 (96.0%)	6246 (96.3%)		Ref
Instrumental delivery	115 (3.5%)	127 (4.0%)	242 (3.7%)		1.13 (0.88, 1.47)
**Gestational age**	38.4 ± 2.2	38.5 ± 2.2	38.4 ± 2.2	0.11	
37–41 week	2558 (79.4%)	2324 (81.1%)	4882 (80.2%)		Ref
< 37 week	580 (18.0%)	487 (17.0%)	1067 (17.5%)		0.92 (0.81, 1.06)
≥ 42 week	82 (2.5%)	54 (1.9%)	136 (2.2%)		0.73 (0.51, 1.03)
**Birthweight**	2887.8 ± 486.4	2883.0 ± 516.4	2885.5 ± 500.7	0.29	
2500–4000 g	2603 (80.8%)	2277 (79.5%)	4880 (80.2%)		Ref
< 2500 g	574 (17.8%)	554 (19.3%)	1128 (18.5%)		1.10 (0.97, 1.26)
≥ 4000 g	43 (1.3%)	34 (1.2%)	77 (1.3%)		0.90 (0.57, 1.42)
**Newborn placed immediately in skin to skin contact with mother**				< 0.001	
No	2621 (80.1%)	2090 (65.1%)	4711 (72.7%)		Ref
Yes	653 (19.9%)	1120 (34.9%)	1773 (27.3%)		2.15 (1.92, 2.41)
**Timing of cord clamping**				< 0.001	
Early (< 3 min)	1825 (64.7%)	1327 (55.5%)	3152 (60.5%)		Ref
Delayed (≥3 min)	997 (35.3%)	1065 (44.5%)	2062 (39.5%)		1.47 (1.31, 1.64)

*cOR* Crude Odds Ratio

In bi-variate logistic regression, multiparous mothers had higher odds for timely initiation of breastfeeding (cOR 1.44; 95% CI 1.28, 1.62) when compared to nulliparous women. A Mother with no obstetric complication at the time of admission had two-fold odds of timely initiation of breastfeeding (cOR 2.04; 95% CI 1.77, 2.35). Neonates when placed skin to skin contact with the mother soon after birth had more than twice-fold higher odds of timely breastfeeding (cOR 2.15; 95% CI 1.92, 2.41). Delayed cord clamped neonates had higher odds of 47% of timely initiation of breastfeeding compared to early cord clamped (cOR 1.47; 95% CI 1.31, 1.64) (Table 2).

In the multivariate logistic regression, multiparous mothers had 56% higher odds of timely initiation of breastfeeding (aOR 1.56; 95% CI 1.35, 1.82). Women with no obstetric complication at the time of admission had 57% increased odds of timely initiation of breastfeeding (aOR 1.57; 95% CI 1.33, 1.86). Neonates born after a gestational age of less than 37 weeks had 19% lower odds of timely initiation of breastfeeding (aOR 0.81; 95% CI 0.66, 0.98) when compared to neonates born 37 weeks or more. Forty-six percent higher odds of timely initiation of breastfeeding were found among birthweight less than 2500 g neonates (aOR 1.46; 95% CI 1.21, 1.76). Neonates when placed skin-to-skin with mother soon after birth had 2.5-fold odds of timely initiation of breastfeeding (aOR 2.52; 95% CI 2.19, 2.89). Neonates who had a delay cord clamped had 37% higher odds of timely initiation of breastfeeding (aOR 1.37; 95% CI 1.21, 1.55) (Table 3).

**Table 3:** Multivariate analysis of factors associated with timely initiation of breastfeeding

Predictors	***p***-value	B-coefficient	aOR (95%CI)
**Parity**			
1 previous birth			Ref
0 previous birth	< 0.001	−0.29	0.75 (0.65, 0.87)
2 or more previous births	< 0.001	0.45	1.56 (1.35, 1.82)
**Obstetric complication**			
Yes			Ref
No	< 0.001	0.45	1.57 (1.33, 1.86)
**Mode of delivery**			
Spontaneous vaginal delivery			Ref
Instrumental delivery	0.47	0.11	1.12 (0.82, 1.53)
**Gestational age**			
37–41 week			Ref
< 37 week	0.03	−0.22	0.81 (0.66, 0.98)
≥ 42 week	0.36	−0.19	0.83 (0.55, 1.24)
**Birthweight**			
2500–4000 g			Ref
< 2500 g	< 0.001	0.38	1.46 (1.21, 1.76)
≥ 4000 g	0.14	−0.42	0.66 (0.38, 1.14)
**Neonates placed immediately in skin to skin contact with mother**			
No			Ref
Yes	< 0.001	0.92	2.52 (2.19, 2.89)
**Timing of cord clamping**			
Early (< 3 min)			Ref
Delayed (≥3 min)	< 0.001	0.32	1.37 (1.21, 1.55)

*aOR* Adjusted Odds ratio

## Discussion

About half of women initiated timely breastfeeding. Multiparity, no obstetric complication at admission, neonates placed in skin-to-skin contact soon after birth and delay cord clamping were strong predictors for timely initiation of breastfeeding.

Delayed cord clamping, a significant predictor for timely initiation of breastfeeding [20], has immediate better physiological outcomes and long-term behavioral outcomes [21]. Improved physiologic stability and alertness immediately after birth could provide major support to successful timely initiation of breastfeeding.

Positive association was found between neonates placed skin-to-skin contact soon after birth and timely initiation of breastfeeding corresponds to the studies that have recommended skin-to-skin contact as crucial step to timely initiation of breastfeeding [4, 22]. Early maternal-infant attachment evokes psycho-physiological behaviors that may be conducive to fulfill basic biological needs [23]. Robust findings and strong recommendations supporting the benefits of early skin to skin contact on timely initiation of breastfeeding have accelerated scale up of quality improvement programs of maternal and newborn care at both national and global levels [14, 24].

Studies have explored the association of maternal parity with timely initiation of breastfeeding [24–27]. Relatively, we also found that nulliparous mothers are unlikely to initiate timely breastfeeding. This finding is similar to the study done in India, in which the first-time mothers were more likely to delay breastfeeding [28]. First-time mothers might not be familiar with the practice of keeping neonates skin-to-skin and timely initiation of breastfeeding.

Timely initiation of breastfeeding was significantly higher among mothers with no obstetric complications at the time of admission to the hospital. Women with obstetric complication require additional intrapartum management restricting neonates to be early breastfed [13]. Neonates born to mothers with an intrapartum complication are at high risk of developing sepsis, hypothermia and hypoglycemia and can possibly benefit from receiving their mother’s breastmilk early [29]. Neonates born after gestational age less than 37 weeks were found to have lower odds of timely initiation of breastfeeding than the full term neonates. These neonates required additional breathing support and require additional support to timely breastfeeding [30]. Increased timely breastfeeding in low birthweight babies could be due to the reinforcement through quality improvement interventions implemented in these hospitals [31].

A major strength of this study is the direct observation of breastfeeding by well-trained observers using a structured checklist in all four hospitals.

### Limitations

There are several limitations to the study. First, some of the sociodemographic information such as maternal ethnicity, age and sex of neonates was not available for analysis. Second, this study was done to provide descriptive data on the timely initiation of breastfeeding and its predictors among the hospital born neonates, however, observation was limited to only vaginal births excluding neonates born through cesarean section. All the four hospitals had variation in vaginal birth rate, hence, the findings should not necessarily be generalizable to all the public hospitals of Nepal. Finally, the Hawthorne effect might have influenced the behavior of healthcare provider underestimating the true prevalence than explored, although efforts were made to reduce the possible effects of observation [32, 33].

## Conclusions

Delayed cord clamping and immediate skin to skin contact are strong predictors for timely initiation of breastfeeding. An institutional birth, a breastfeeding support program focusing on nulliparous mothers and those with obstetric complication, can increase the timely initiation of breastfeeding. A pregnant woman needs emotional and physical support throughout pregnancy, childbirth and postnatal period. A confident and happy mother who receives continuous support from the healthcare provider can be successful in timely initiation of breastfeeding. Quality improvement interventions can improve the practice through accountability and leadership. Further research is required to understand the factors associated with timely initiation of breastfeeding practice and develop an effective intervention.

## Data Availability

The dataset generated and analysed is not publicly available as it is part of larger quality improvement project but can be made available on reasonable request with a data-sharing agreement.

## Abbreviations

aOR: Adjusted Odds Ratio
CB-NCP: Community Based Newborn Care Package
CSPro: Census and Survey Processing System
LMIC: Low and Middle Income Countries
SPSS: Statistical Package for Social Sciences
